# Usage of Inhalative Sedative for Sedation and Treatment of Patient with Severe Brain Injury in Germany, a Nationwide Survey

**DOI:** 10.3390/jcm12196401

**Published:** 2023-10-08

**Authors:** Svea Roxana Roggenbuck, André Worm, Martin Juenemann, Christian Claudi, Omar Alhaj Omar, Marlene Tschernatsch, Hagen B. Huttner, Patrick Schramm

**Affiliations:** Department of Neurology, University Hospital of the Justus-Liebig-University Giessen, Klinikstrasse 33, 35392 Giessen, Germany; svearoxana.roggenbuck@neuro.med.uni-giessen.de (S.R.R.); andre.worm@neuro.med.uni-giessen.de (A.W.); martin.juenemann@neuro.med.uni-giessen.de (M.J.); christian.claudi@neuro.med.uni-giessen.de (C.C.); omar.alhajomar@neuro.med.uni-giessen.de (O.A.O.); marlene.tschernatsch@neuro.med.uni-giessen.de (M.T.); hagen.huttner@med.jlug.de (H.B.H.)

**Keywords:** surveys and questionnaires, critical care, inhalative anesthetics, inhalative sedation, isoflurane, sevoflurane, brain injury, neuromonitoring

## Abstract

Brain injured patients often need deep sedation to prevent or treat increased intracranial pressure. The mainly used IV sedatives have side effects and/or high context-sensitive half-lives, limiting their use. Inhalative sedatives have comparatively minor side effects and a brief context-sensitive half-life. Despite the theoretical advantages, evidence in this patient group is lacking. A Germany-wide survey with 21 questions was conducted to find out how widespread the use of inhaled sedation is. An invitation for the survey was sent to 226 leaders of intensive care units (ICU) treating patients with brain injury as listed by the German Society for Neurointensive Care. Eighty-nine participants answered the questionnaire, but not all items were responded to, which resulted in different absolute counts. Most of them (88%) were university or high-level hospital ICU leaders and (67%) were leaders of specialized neuro-ICUs. Of these, 53/81 (65%) use inhalative sedation, and of the remaining 28, 17 reported interest in using this kind of sedation. Isoflurane is used by 43/53 (81%), sevoflurane by 15/53 (28%), and desflurane by 2. Hypotension and mydriasis are the most common reported side effects (25%). The presented survey showed that inhalative sedatives were used in a significant number of intensive care units in Germany to treat severely brain-injured patients.

## 1. Introduction

Patients with severe brain injury due to acute neurological or neurosurgical illnesses often need deep sedation to prevent and/or reduce increased intracranial pressure (ICP) [1,2]. A phase of deep sedation for the first part of the intensive care unit (ICU) treatment is followed by an awakening phase. Long-acting pharmaceuticals should not delay this phase to allow a more precise prognosis and early rehabilitation. Therefore, the best sedative for patients with severe brain injury should allow for deep sedation with minimal side effects and a short context-sensitive half-life for rapid awakening. The current recommendation, propofol as an IV sedative, has a short context-sensitive half-life and few side effects [3]. Nevertheless, there is a life-threatening side effect: propofol infusion syndrome (PRIS). PRIS incidence is higher in younger patients and increases with a higher dose of propofol, but it can also occur in older patients with a lower dose [4]. Consequently, the likelihood of extended deep sedation with propofol is restricted by time and dosage, and the sedation protocol is often supplemented with benzodiazepines or switched to benzodiazepines entirely. Benzodiazepines have a very high context-sensitive half-life and significantly extend awakening [3]. Furthermore, benzodiazepines increase the risk of delirium and prolonged ventilation time [5]. Possible alternative sedatives are inhalative anesthetic drugs such as isoflurane and sevoflurane. These drugs are easy to control, are not or very little metabolized, and have a brief context-sensitive half-life. Additionally, deep sedation with moderate side effects is possible [6,7,8]. In general intensive care patients, isoflurane is safe and shortens the time of awakening and extubation [9,10]. Thus, isoflurane seems to live up to the claim of being the best sedative for severely brain-injured patients, but no study has examined this. To evaluate the current clinical usage of inhalative sedatives in treating patients with severely injured brains, we have conducted a national survey in Germany.

## 2. Materials and Methods

The survey was created as a cross-sectional online survey conducted between June and October 2022 using LimeSurvey^®^ (version 3.23.1, LimeSurvey GmbH, Hamburg, Germany) software, adhering to the published Consensus-Based Checklist for Reporting of Survey Studies (CROSS) [11].

The local ethics committee of the Justus-Liebig-University Giessen, Germany, approved the survey. A new questionnaire with 21 items was created for this survey with two sections. In the first section, 7 items address the baseline characteristics of the hospital and the ICU. The second section deals with the main point of the survey: the use of inhalative sedatives in the participant’s intensive care unit. If the participant stated that they did not use inhalative sedatives, two other items address the potential interest in using these agents and why they do not use inhalative sedatives. If the participant stated that they use inhalative sedatives, ten additional items address the kind of inhalative sedative, application pathway, difficulties and side effects, indications, contraindications, and the applied neuromonitoring. Single and multiple answer questions were included.

The first version of the questionnaire was reviewed and revised by SR, AW, PS, and OA. Additionally, MJ, HBH, MT, and CC, who had not drafted the first version, pre-tested the questionnaire afterward. Building upon these reviews, we developed the final questionnaire, which was reviewed and approved by all authors. All questionnaire items, including the structure and answers, will be provided in a translated version as additional file. The survey was addressed to all leaders of the ICUs listed by the German Society for Neurointensive Care and Neuro Emergency Medicine (DGNI, Deutsche Gesellschaft für NeuroIntensiv- und Notfallmedizin) as treating patients with brain injuries. These ICUs are either part of the neurological or neurosurgical department or interdisciplinary under the direction of the department for anesthesiology or internal medicine. The survey was sent to the heads of the ICU after an online check was carried out to determine whether the contact persons were still correct. If this was not possible, the head of the neurologic and/or neurosurgical department was addressed. This updated list containing the email addresses was used for the individual invitation to participate in this survey. Participation in the survey depended on an automatically generated invitation by the LimeSurvey^TM^ program linked with the email address. In this way, an invited participant could only answer this survey once. The first invitation was sent in June 2022, and a second reminder was sent in October 2022 (the latter did not address the participants who had already answered). No personal data were collected in the survey, and backtracking was impossible. The survey data were collected on a German password secured server.

Statistical analysis was descriptive and performed with IBM SPSS Statistics (Version 24, IBM Corp., Armonk, NY, USA) and MS-Excel (Microsoft, Redmond, WA, USA). Data are presented as sum and percent.

## 3. Results

The invited 226 ICUs from the list of the DGNI were mostly specified as interdisciplinary (154/226), treating patients with brain injuries but not specialized in this collective of patients. Of the remaining ICUs, 51 were declared as specialized neurological and 21 as specialized neurosurgical ICUs. For each ICU, only one participant was invited, either the leading physician or the head of the department. After the first invitation, 59 questionnaires were returned, and 89 were received after the reminder five months later. Not all items of the dataset were answered by all participants, which resulted in different absolute counts. The whole questionnaire, including all items, the question flow, and the detailed answers, is provided as additional file 1.

### 3.1. Participants

**Figure 1:**
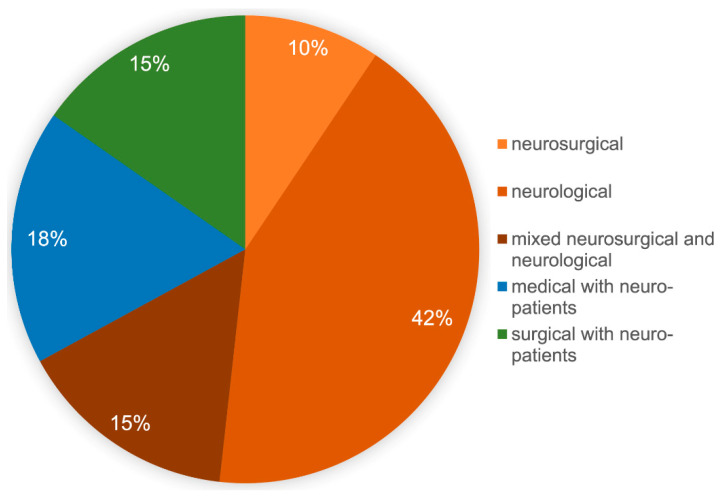
Type of ICU as stated by the participants of the survey. Allocation of the participants of the survey to their primary profession of the ICU.

In order to better assess the results, a description of the responding participants is first provided. Of the returned 89 data sets, 2 participants did not answer the question about the care-level of their own hospital own hospital, 46/87 (53%) were from university hospitals, 29/87 (33%) were from high-level hospitals, and 12/87 (14%) were from primary care hospitals. The form of ICU was neurosurgical in 8/85 (9%), neurological in 36/85 (42%), and mixed neurosurgical and neurological in 13/85 (15%). Furthermore, 15/85 (18%) described their ICU as an internal medicine ICU and 13/85 (15%) as an anesthesiologic/surgical ICU. Each declared the treatment of severely brain-injured patients (Figure 1); 4 participants did not answer the question about their own ICU.

Based on the list of the DGNI, in Germany there are 51 specialized neurological intensive care units and 21 specialized neurosurgical intensive care units. Of the 51 specialized neurological intensive care units, 42/51 (83%) participants answered the questionnaire, while 8 of the 21 specialized neurosurgical intensive care units (38%) did so. From the remaining 154 ICUs from the list of the DGNI, 48 (32%) responded to the survey and provided detailed information, dividing these into 15 mixed neurological and neurosurgical ICUs, 18 internal medicine, and 15 anesthesiologic/surgical ICUs. At the same time, the latter two stated that they treat neurological and/or neurosurgical patients.

The last question about the characterization and classification of the ICU concerned the size of the ward. Six participants did not answer this question, while 44 of the 83 (53%) participants specified that they had more than 12 beds, 38 of the 83 (46%) stated that they have 8–12 beds, and 1 ICU had less than 8 ICU beds.

### 3.2. Use of Inhalative Sedatives

**Figure 2:**
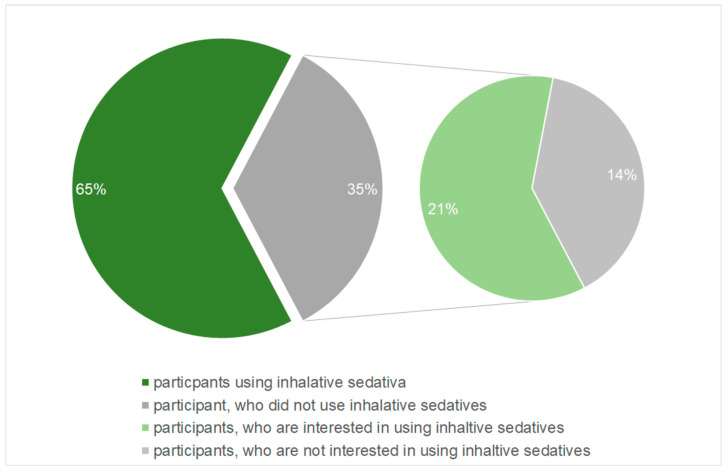
Distribution of user behavior and interest in usage of inhalative sedatives. Of the 81 participants who responded to the main question, 53 said they use anesthetic gases, and of the remaining 28, 17 said they were interested in using them.

The main question of the survey asked if the participants use inhalative sedatives in the treatment of brain-injured patients. This was answered by 81 of the participants. Of these 81 participants, 52 (65%) answered the question whether they used inhalative sedatives with yes. The remaining 28 (35%) participants, who negated the use of inhalative sedatives, were asked if they are principally interested in using this kind of sedation. Of the 28 participants who did not use inhalative sedatives, the majority, 17 respondants (61%), indicated that they had a potential interest in using inhalative sedatives (Figure 2).

**Figure 3:**
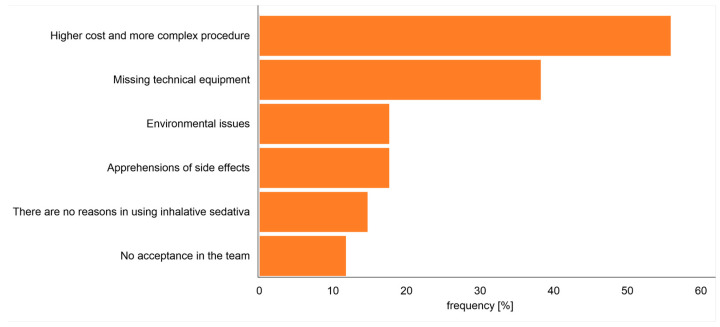
Reasons of the participants who did not use inhalative sedatives. The figure shows the reasons for not using inhalative sedatives. Multiple answers were possible.

Following whether there existed a principal interest in using inhalative sedatives, the participants not using this kind of sedation were asked about the main reason for not using inhalative sedatives. Most of the participants (19/28; 68%) stated the absence of technical equipment as a reason for not using inhalative sedatives. Further given reasons for not using inhalative sedatives were answered as follows: six (21%) participants stated that inhalative sedatives are not helpful, and a further six (21%) participants stated the absence of willingness in the ICU team. Five (18%) of these participants stated the fear of side effects (not specified in more detail by this survey). About half (47%) of the answered participants stated higher costs and extra efforts as a reason for not using inhalative sedatives and four (14%) participants stated negative environmental impact by the drug. Multiple answers were possible (Figure 3).

### 3.3. Kind of Inhalative Sedative

After considering the reasons for not using inhalative sedatives, we present the detailed answers of the participants using inhalative sedatives. Of the 53 participants using inhalative sedatives, 43 (81%) used isoflurane, 15 (28%) sevoflurane, and 2 desflurane, with more than one answer possible. The reasons for choosing one or the other inhalative sedative were not recorded.

Generally, there are two different methods to apply inhalative sedatives in the ICU. The use of an anesthetic machine from the operation theatre is not rational for long term sedation. Most of the participants use the Sedaconda™/Anaconda™ (Sedana Medical, Stockholm, Sweden) device (88%) and seven use the Mirus™ (TIM Medical, Koblenz, Germany) device (12%). Both methods used to apply inhalative sedatives need additional technical equipment, so the next two questions address the number of devices for application of inhalative sedatives at the individual ICU and whether the participant is satisfied with the number of devices. In the median, the participants stated that they have 2.5 devices for inhalative sedatives [Range 1 to 12]. Of the participants using inhalative sedatives, 37/51 (73%) said that they are content with the number of devices they have available with an affirmative, while 14/51 (27%) would like to have more devices.

### 3.4. Side Effects

Side effects limit the use of drugs. In order to get an impression of which side effects were observed by the users, the participants were asked to share their observations. This was done in free text field without any specifications. Thirteen (25%) participants who use inhalative sedatives observed complications while using inhalative sedatives, while 40/53 (75%) did not observe relevant side effects. The side effects reported by the participants who observed side effects were: hypotension and mydriasis in five answers, ICP elevation in 2, paCO_2_ elevation in 2, gut dysfunctions in 2, and 2 stated technical difficulties using the Anaconda™ device.

### 3.5. Indication and Contraindication

Due to the absence of recommendations for using inhalative sedatives in the case of severely brain injured patients, the participants were asked two questions concerning whether they have specific indications or specific contraindications for or against using inhalative sedatives. Among the participants using inhalative sedatives, 21/53 (40%) stated that they have no specific indications for its application. The individual clinical pictures for using inhalative sedatives by the remaining 32 participants are listed in Table 1. Further, 26 out of 53 (49%) said they had no contraindications for using inhalative sedatives. The excluded illnesses for the remaining 27 participants are listed in Table 2.

### 3.6. Neuromonitoring and Monitoring Depth of Sedation

Specialized neuromonitoring is often used in the treatment of brain injured patients. As such, the next questions asked if the participants use neuromonitoring combined with the use of inhalative sedatives, and if so, which kind they used. Specialized neuromonitoring was used by 48 of the 53 participants, but 20 of these 48 stated that this was not mandatory. The applied neuromonitoring technics are listed in Table 3.

The final question to the participants using inhalative sedation deal with monitoring of the depth of sedation. The depth of sedation was declared to be measured in 21 of 51 answers using processed EEG (automatic measurement of the depth of anesthesia), in 19 by raw-EEG and interpretation by the ICU staff, and in 44 by clinical parameters such as the Richmond Agitation and Sedation Scale (RASS) [12]. More than one answer was possible.

## 4. Discussion

This survey showed that the application of inhalative sedation for severely brain-injured patients is used by a significant number of neurological, neurosurgical, anesthesiologic, and internal medicine ICUs in Germany. Furthermore, most participants who were not using inhalative sedatives were interested in using them. Isoflurane is used by most, followed by sevoflurane. Only ¼ of the participants using inhalative sedatives observed side effects; none reported life-threatening side effects. The indication spectrum includes neurological and neurosurgical diseases, ARDS (acute respiratory distress syndrome), and sepsis.

### 4.1. Participants

The directory of ICUs that care for brain-injured patients is a voluntary list of hospitals and ICUs by the DGNI and not an official list by the government or the German hospital federation. Most of them are stated as interdisciplinary and are in primary care hospitals. Of these, the neurological or neurosurgical department head was listed despite not leading the ICU by themself. It can therefore be assumed that many of the people contacted are not involved in the care of severely brain-injured patients and, for this reason, did not answer the survey. By far, the most responses came from high-level or university hospitals with more than eight ICU beds in the individual unit. It can therefore be assumed, despite the average return of responses, that a significant number of the relevant hospitals that treat severely brain-injured patients responded to the survey.

### 4.2. Use of Inhalative Sedatives

The present survey suggests a significant use of inhalative sedatives in Germany in the treatment of severely brain-injured patients, independent of the primary profession of intensive care physicians. These findings contrast with a French observational study that observed the kind of sedatives used in a one-day observation of the sedation management in brain-injured patients at 30 ICUs in France, where none of the patients were treated with inhalative sedatives [13]. This might be a random effect by investigating only one day because another French survey with 187 participating ICUs reported using inhalative sedatives in a mixed collective but also brain-injured patients [14]. The authors did not find additional publications in other countries about using inhalative sedatives at the ICU, especially not in brain-injured patients. However, the current survey and the French survey showed that inhalative sedatives were used despite the lack of large comparative randomized trials. Therefore, such comparative trials, especially regarding the theoretical superiority of IV sedatives, are necessary and justified.

### 4.3. Kind of Inhalative Anesthetic

Participants of the survey mostly use isoflurane as an inhalative sedative for brain-injured patients. This coincides with the current study situation. Investigating a diverse collective of 19 patients with cerebrovascular diseases, isoflurane was safe regarding ICP with a well-known lowering effect on the mean arterial pressure (MAP) and cerebral perfusion pressure [7]. The lowering of MAP is inherent in all sedatives to varying degrees and can be treated with vasoconstricting drugs. In a subsequent investigation, also in a diverse collective of 25 patients with cerebrovascular diseases, the application of sevoflurane led to a relevant increase in ICP and to a necessary premature stop in the application of sevoflurane in 8 of these patients [6]. This study establishes the benefit of isoflurane compared to sevoflurane in patients with cerebrovascular diseases.

It is reflected in the results of this survey, with isoflurane being the most used sedatives. In a cross-over designed investigation with 13 patients with subarachnoidal hemorrhage, the treatment with isoflurane for one hour compared to propofol led to an increase of cerebral blood flow while the ICP remained constant [8]. Despite the small number of patients in these studies, isoflurane seems safe in patients with potentially elevated ICP. In addition to the safe use in patients with cerebrovascular diseases, the treatment with isoflurane in patients with refractory status epilepticus was safe and efficient in a retrospective investigation [15]. The broader use of isoflurane is further supported as it is licensed in Europe, and sevoflurane might be potentially nephrotoxic [9,16].

Most of the participants stated that they use the Sedaconda™, previously named Anaconda™, device from Sedana Medical (Stockholm, Sweden) for application of inhalative sedatives. This device has been available since the beginning of the 20 century and, in combination with isoflurane, is authorized for the sedation of ICU patients. In contrast, the certified MIRUS™ controller (TIM GmbH, Koblenz, Germany) was introduced in 2013. Both systems are based on a different handling and require different technical equipment [9,17]. There was no question in the survey about the reason why either device was used. It can be assumed that all participants who use inhalative sedatives have been familiar with the use of inhalation sedation for a longer time-period and therefore use the device that has been available longer.

Not addressed by the survey was the scavenging system used to avoid air pollution. While in the operating theatre a special scavenging system for inhalative sedatives exists, at the ICU this is not always available. If such a specialized scavenging system at the ICU is not available, a special activated carbon filter is used to clear the exhaled air after the respirator.

### 4.4. Side Effects

Side effects of sedatives, IV or inhalative, are common, especially in the context of critically ill patients. In the current survey, the main reported side effect of using inhalative sedatives was hypotension and mydriasis. Although vasopressors are effective in treating blood pressure reductions, such as norepinephrine, mydriasis can lead to unnecessary computed tomography (CT), increased workload, exposure to X-rays, and potential damage caused by transport to the radiological theatre. The actual incidence of pupil motoric disorder is not known and was reported in less than 10% of the participants of the current survey. The knowledge of this side effect may be helpful to indicate CT scans in this situation critically, and the indication may be supported by multimodal neuromonitoring. The reported increase of paCO_2_ is explicable by the additional ventilatory death space due to the application device and may be treated with an adapted higher tidal volume. In summary, the reported side effects are of low incidence and the use of isoflurane may be safe in brain-injured patients.

Interestingly, none of the participants report malignant hyperthermia as an observed or anticipated side effect. Malignant hyperthermia is a rare but life threatening side effect in case of genetical predisposition and contact to inhalative sedatives. It is recommended to establish a standard operating procedure for early recognition of the clinical signs of malignant hyperthermia and promptly stop the exposition and initiate treatment. In this survey, the participants were not asked if they have this recommendation implemented and therefore, no statement is possible. The authors of this article already have such a standard operating procedure and recommend this for all ICU who use or want to use inhalative sedatives.

### 4.5. Indication and Contraindication

There are no clear recommendations for using different sedatives according to different brain injuries. Furthermore, no confirmatory trials investigated different sedatives in brain injury regarding outcome. This absence of evidence is reflected in the given answers of this survey. Half of the participant state that they have no specific indication or contraindication for using inhalative sedatives. The remaining participants stated specific illnesses as indicated or contraindicated, which, in the end, seems rather coincidental and is more likely to be based on individual pathophysiological assessment and experience.

### 4.6. Neuromonitoring and Monitoring the Depth of Sedation

Neuromonitoring is routinely used in ICUs that treat brain-injured patients. This is reflected in the answers to the current survey, although many participants did not use neuromonitoring exclusively for inhalation sedatives. Monitoring the depth of sedation is recommended to ensure the lowest level of sedation necessary is achieved to prevent over-sedation [3]. This should be either performed by clinical investigation, for example, using the RASS for mild sedation, or by EEG and EEG-derived indices for depth of anesthesia for patients needing deep sedation [3,12,18]. Especially for patients with severe brain injury and potential focal or global increase of ICP, deep sedation is recommended to reduce brain activity and, therefore, cerebral blood volume and ICP [2]. This effect of lowering ICP is limited to reducing brain activity to a burst-suppression EEG, while additional sedation may result in increased side effects without additional impact on ICP [3,18]. The knowledge and implementation of this necessary targeted sedation control were known among the participants in this survey, which was reflected in more than 75% use of EEG and EEG-derived monitoring. However, clinical monitoring using the RASS was also reported by most participants, during the mild sedation phase. Neuromonitoring is widely used by all participants, especially for measuring the depth of sedation.

## 5. Conclusions

The national online survey showed that inhalative sedatives are used by a significant number of relevant hospitals in Germany when treating severely brain-injured patients. The response rate was moderate but focused on high-level hospitals and, therefore, those centers that primarily treat these patients. Of the participants who responded, 65% used inhalative sedatives, mainly isoflurane, and only 25% reported side effects. These results underline the usefulness of studies to further investigate the value of inhalative sedatives compared to IV sedatives.

## Figures and Tables

**Table 1:** Diseases, participants stated as specific indications for using inhalative sedation.

Disease	Frequency [n]
No specific indication	21
Status epilepticus	32
Sepsis	21
Intracerebral hemorrhage	18
Subarachnoidal hemorrhage	17
ARDS	15
Cerebral ischemia	14
Neuroimmunologic disease	11
Brain trauma	10
Postoperative sedation	3

Answers to the question: “For which clinical indications do you use inhalative anesthetics?” of all participants who use inhalative anesthetics (n = 53). Multiple answers were possible. ARDS: acute respiratory distress syndrome.

**Table 2:** Diseases participants stated as specific contraindications for inhalative sedation.

Disease	Frequency [n]
No specific disease	26
Brain trauma	11
Cerebral ischemia	8
ARDS	8
Sepsis	8
Intracerebral hemorrhage	7
Postoperative sedation	7
Subarachnoidal hemorrhage	5
Neuroimmunologic disease	3
Status epilepticus	2

Answers to the question: “For which clinical indications do you not use inhalative anesthetics?” of all participants who use inhalative anesthetics (n = 53). Multiple answers were possible. ARDS: acute respiratory distress syndrome.

**Table 3:** Specific neuromonitoring techniques while using inhalative sedatives.

Neuromonitoring Technique	Frequency [n]
EEG	24
ICP measurement	17
processed EEG	11
NIRS	7
ptiO_2_ measurement	5

Answers to the question: “Which kind of monitoring is obligatory while using inhalative sedatives?” of all participants who use inhalative anesthetics and further stated that they use specific neuromonitoring (n = 28). Multiple answers were possible. EEG: electroencephalography, ICP: intracranial pressure, NIRS: near infrared spectroscopy, ptiO_2_: brain tissue oxygen pressure.

## Data Availability

The presented data are part of the doctoral thesis of Mrs. Svea Roxana Roggenbuck. All data generated or analyzed during this study are included in this published article and its Supplementary Information Files.

## Supplementary Materials

The following supporting information can be downloaded at: https://www.mdpi.com/article/10.3390/jcm12196401/s1, IsoSurvey–Questionnaire and detailed answers: IsoSurvey appendix.docx.
