# Supplementary figures and images for: Usage of Inhalative Sedative for Sedation and Treatment of Patient with Severe Brain Injury in Germany, a Nationwide Survey
Source: J Clin Med. 2023 Oct 8;12(19):6401. doi: 10.3390/jcm12196401 (PMC10573088; doi:10.3390/jcm12196401)

## IsoSurvey – Questionnaire

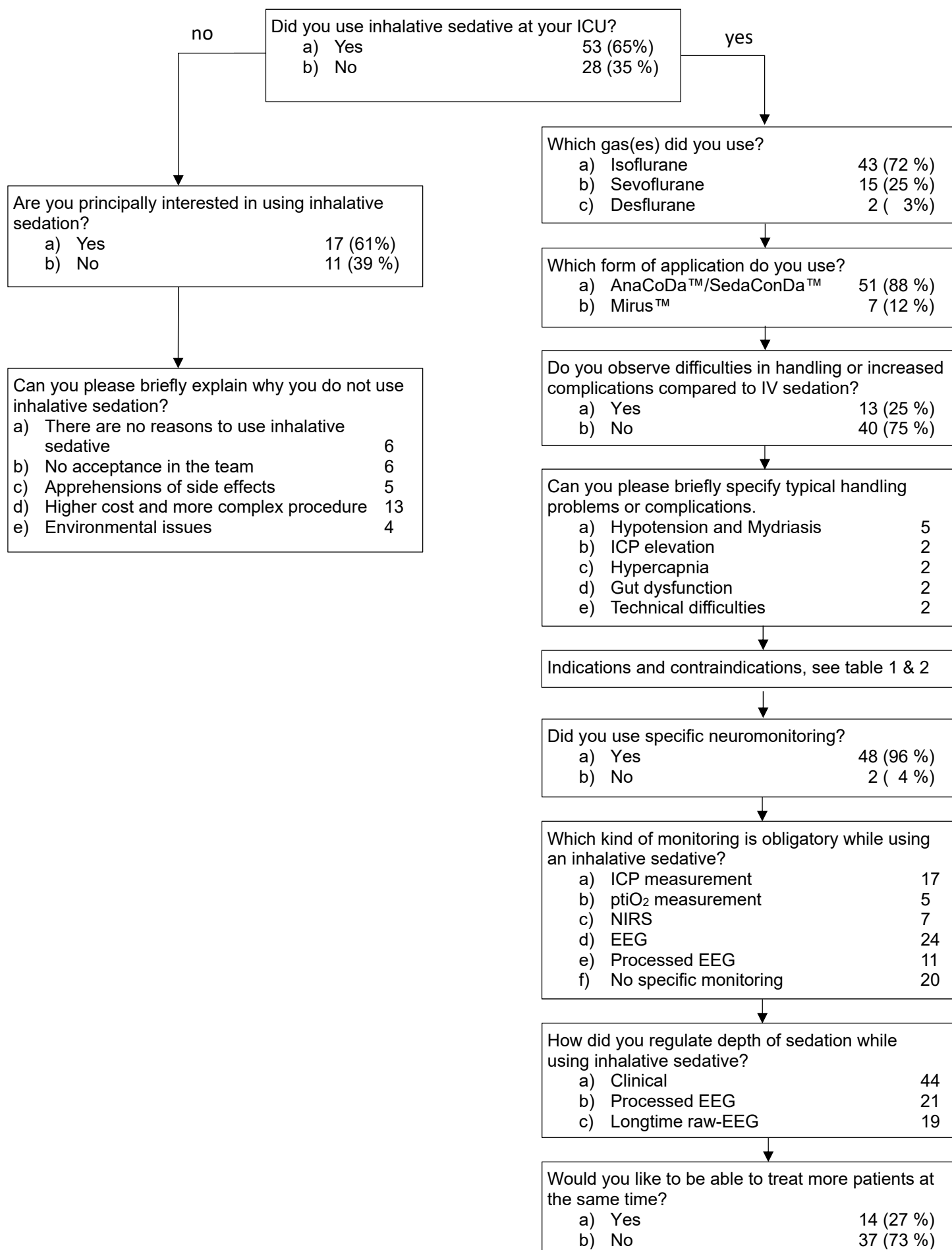

Supplement: Supplementary file 1 [file jcm-12-06401-s001.zip › jcm-2600055-supplementary.pdf]
